# Iron oxide nanoparticles induce human microvascular endothelial cell permeability through reactive oxygen species production and microtubule remodeling

**DOI:** 10.1186/1743-8977-6-1

**Published:** 2009-01-09

**Authors:** Patrick L Apopa, Yong Qian, Rong Shao, Nancy Lan Guo, Diane Schwegler-Berry, Maricica Pacurari, Dale Porter, Xianglin Shi, Val Vallyathan, Vincent Castranova, Daniel C Flynn

**Affiliations:** 1 The Pathology and Physiology Research Branch, Health Effects Laboratory Division, National Institute for Occupational Safety and Health, Morgantown, WV 26505, USA; 2 MBR Cancer Center, School of Medicine, West Virginia University, Morgantown, WV 26506-9300, USA; 3 Pioneer Valley Life Sciences Institute, Baystate Medical Center/University of Massachusetts at Amherst, Springfield, MA 01107, USA; 4 MBR Cancer Center/Department of Community Medicine, School of Medicine, West Virginia University, Morgantown, WV 26506-9300, USA; 5 Department of Physiology and Pharmacology, School of Medicine, West Virginia University, Morgantown, WV 26506, USA; 6 The Commonwealth Medical College, Scranton, PA 18510, USA

## Abstract

**Background:**

Engineered iron nanoparticles are being explored for the development of biomedical applications and many other industry purposes. However, to date little is known concerning the precise mechanisms of translocation of iron nanoparticles into targeted tissues and organs from blood circulation, as well as the underlying implications of potential harmful health effects in human.

**Results:**

The confocal microscopy imaging analysis demonstrates that exposure to engineered iron nanoparticles induces an increase in cell permeability in human microvascular endothelial cells. Our studies further reveal iron nanoparticles enhance the permeability through the production of reactive oxygen species (ROS) and the stabilization of microtubules. We also showed Akt/GSK-3β signaling pathways are involved in iron nanoparticle-induced cell permeability. The inhibition of ROS demonstrate ROS play a major role in regulating Akt/GSK-3β – mediated cell permeability upon iron nanoparticle exposure. These results provide new insights into the bioreactivity of engineered iron nanoparticles which can inform potential applications in medical imaging or drug delivery.

**Conclusion:**

Our results indicate that exposure to iron nanoparticles induces an increase in endothelial cell permeability through ROS oxidative stress-modulated microtubule remodeling. The findings from this study provide new understandings on the effects of nanoparticles on vascular transport of macromolecules and drugs.

## Background

Iron nanoparticles are of great interest due to their unique physicochemical properties and have been used for the development of imaging, magnetic and electrical applications [1]. Recently, iron nanoparticles have been widely used in coal industry to produce clean fuels due to their catalytic activities that facilitate the chemical reactions to form and cleave carbon-carbon bonds [2]. More importantly, iron nanoparticles show great potential in human biomedical applications, such as labeling and magnetic separation of biological materials, imaging and diagnostic applications in human, site-directed drug delivery, and anticancer hyperthermia therapy [2]. However, significant knowledge gaps currently exist on the precise mechanisms of translocation of iron nanoparticles into the targeted tissues, organs, and tumors, as well as the toxicological effect of iron nanoparticles, which would deter their broad applications.

Endothelial cells play a central role in angiogenesis, carcinogenesis, atherosclerosis, myocardial infarction, limb and cardiac ischemia, and tumor growth [3,4]. The endothelium is an important target for drug and gene therapy. The vascular endothelial monolayer forms a semi-selective permeability barrier between blood and the interstitial space to control the movement of blood fluid, proteins, and macromolecules across the vessel wall. Alteration of permeability barrier integrity plays a major role in drug-based therapies, as well as the pathogenesis of cardiovascular diseases, inflammation, acute lung injury syndromes, and carcinogenesis [3,5,6].

Several studies have shown that intravenously administrated iron nanoparticles can translocate from the blood circulation into various targeted tissues and organs [1,7]. However, it is not clear how iron nanoparticles cross the endothelium from the blood stream into the targeted sites. In this study, we sought to examine whether iron nanoparticle exposure would induce an increase in permeability in human microvascular endothelial cells (HMVECs) and to determine the underlying molecular mechanisms involved. Particular emphasis was focused on the involvements of iron nanoparticle-induced reactive oxygen species (ROS) production in endothelial cell permeability changes. The results in this report demonstrate that iron nanoparticle exposure induces an increase in permeability in HMVECs. This iron nanoparticle-induced permeability involves the production of ROS and the stabilization of microtubules. Furthermore, it was found that PI-3 kinase/Akt/GSK-3β pathways are the important mediators for iron nanoparticle-induced endothelial cell permeability. The results obtained from this study provide the evidence, for the first time, showing that iron nanoparticles may cross the endothelial monolayer through the induction of cell permeability. The results obtained from this study may also provide some insights for understanding the translocation pathways of nanoparticles in general.

## Results

### Size distribution of nanoparticle in cell culture medium and uptake of iron nanoparticles by HMVECs

**Figure 1:**
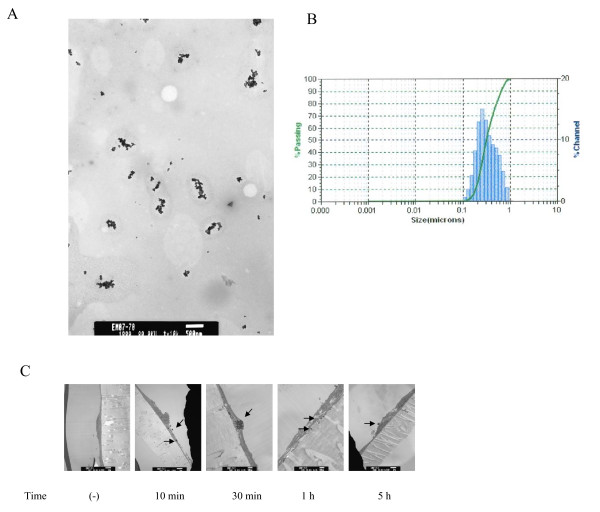
**Size distribution and cell uptake of iron nanoparticles**. A, TEM micrograph of iron nanoparticles in 0.1% FBS cell culture media at the concentration of 50 μg/ml. B, Dynamic light scattering measurements of iron nanoparticles in 0.1% FBS cell culture media at the concentration of 50 μg/ml. C, TEM micrograph of iron nanoparticle uptake by HMVECs. HMVECs were stimulated with 50 μg/ml iron nanoparticles for different periods of time ranging from 10 minutes to 5 hours as indicated. The arrows indicate the location of iron nanoparticles. Each TEM micrograph represents one HMVEC.

Iron nanoparticles used in these experiments are ferrites of maghemite (Fe_2_O_3_), which are superparamagnetic nanoparticles. Unmodified nanoparticles are usually colloidal in nature and prone to agglomerate in suspension [8]. In order to accurately measure the size and distribution of iron nanoparticles in aqueous solutions, a TEM was applied to profile iron nanoparticles in 0.1% FBS cell culture medium. As shown in Figure 1A, the nanoparticles ranged in size from 50 nm-600 nm. Since a TEM can only measure very limited number of particles in solution and the particles subjected to measurements are fixed and dried, it may not provide an accurate profile of the particles in the working solution. Therefore, we applied a dynamic light scattering (DLS) measurement to further characterize the particle size in the working solution. These measurement results showed that iron nanoparticles existed in a size range from 100 nm-700 nm with a mean diameter of 298 nm (Figure 1B). These results demonstrate that iron nanoparticles form small agglomerates, which are uniformly distributed in cell culture medium.

Previously, it was shown that iron nanoparticles can be taken up by mouse macrophages *in vivo *[9] and rat pheochromocytoma cell line (PC12M) *in vitro *[10]. Here, we investigated the uptake of iron nanoparticles by HMVECs. The HMVEC line used here was immortalized by engineering human telomerase catalytic protein (hTERT) into the cells and are therefore able to maintain the inherent features of primary endothelial cells [11]. The cells were cultured to a confluent monolayer on transwell tissue culture-treated polycarbonate membrane polystyrene plates (Corning, NY), and then were stimulated with 50 μg/ml iron nanoparticles for different periods of time ranging from 10 min to 5 h. After the stimulation, the cells were processed for TEM analysis. As shown in figure 1C, the uptake of nanoparticles by HMVECS occurred as early as 10 min after the exposure, and the particles were localized within the cytoplasm of the cells. Approximately, 60% of the cells engulfed the nanoparticles within 30 min after the stimulation (data not shown). The iron nanoparticles were gradually expelled out of the cells, with only 10% of the cells retaining the nanoparticles after 1 h stimulation (data not shown). The results demonstrate that the uptake of iron nanoparticles by HMVECs is both efficient and dynamic.

### Iron nanoparticles induce an increase in permeability of HMVECs

**Figure 2:**
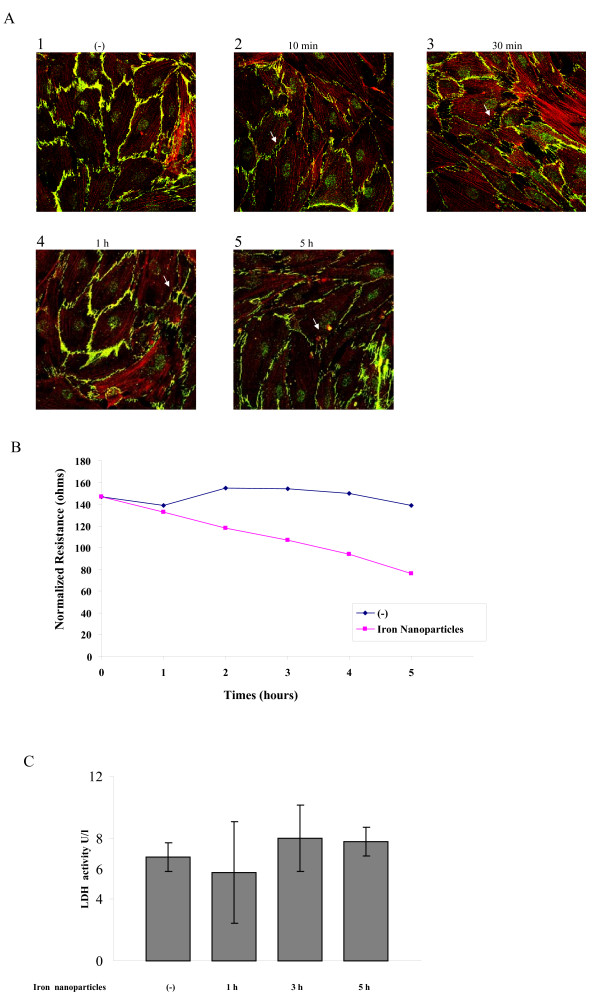
**Iron nanoparticles induce endothelial cell permeability in HMVECs**. A. HMVECs were grown to a confluent monolayer on coverslides and serum-starved overnight. The cells were exposed to 50 μg/ml iron nanoparticle for different periods of time as indicated. After exposure, the cells were fixed, permeabilized, and stained with VE-cadherin (green color) and actin filaments (red color). A Zeiss confocal microscope was applied to take the images. Each image is an overlay of two different stains. Arrows indicate the gaps. B. HMVECs were grown to a confluent monolayer on gold microelectrodes and serum-starved overnight. The cells were treated with 50 μg/ml iron nanoparticles, followed by measuring the transendothelial resistance (TER) for 5 hours. The results shown are representative of 3 independent experiments. C. Exposure of HMVECs to 50 μg/ml iron nanoparticles does not induce cytotoxicity. HMVECs were exposed to 50 μg/ml for different periods of time as indicated. At the end of each exposure, the cell culture media was collected and measured for lactate dehydrogenease (LDH) activities. Values given are means ± SD (t-test, n = 5, p > 0.19).

The changes in endothelial cell permeability not only play a major role in the pathogenesis of cardiovascular diseases, inflammation and cancer, but also have a critical effect on drug delivery to underlying cells, tissues, and organs [12]. We investigated whether exposure to iron nanoparticles would induce an increase in endothelial cell permeability. These results using confocal microscopy image analysis show that the unstimulated HMVECs were attached to each other tightly with no significant intercellular gaps in the HMVEC monolayer (Figure 2A1). However, upon exposure to iron nanoparticles, the confluent monolayer was pulled apart and the cells were separated from each other to form intercellular gaps, which is a hallmark of cell permeability increase [3]. The permeability increase occurred as early as 10 min after the exposure and persisted up to 5 h (Figure 2A2–5). The increase in permeability peaked around 30 min after the exposure (Figure 2A3). To further prove iron nanoparticle-induced HMVEC permeability, we measured transendothelial electrical resistance (TER) across HMVEC monolayer with an electric cell-substrate impedance sensor (ECIS). Our results demonstrate that exposure of HMVECs to iron nanoparticles decreased electrical resistance (Figure 2B), indicating endothelial monolayer barrier compromise. Our dose-dependent ECIS assays demonstrate that iron nanoparticles have an ability to induce endothelial permeability at the concentrations ranging from 12.5 μg/ml to 100 μg/ml (data not shown). To rule out the possibility that iron nanoparticle may induce endothelial permeability change due to cytotoxicity-related cell damage, the LDH (lactate dehydrogenase) release assays were performed. Our results indicate that at the concentration of 50 μg/ml, iron nanoparticles did not significantly induce cytotoxicity within 5 hours of incubation (Figure 2C). Taken together, these results demonstrate that iron nanoparticles have an ability to induce an increase in cell permeability in HMVECs.

### Iron nanoparticles induce cell permeability through microtubule remodeling in HMVECs

**Figure 3:**
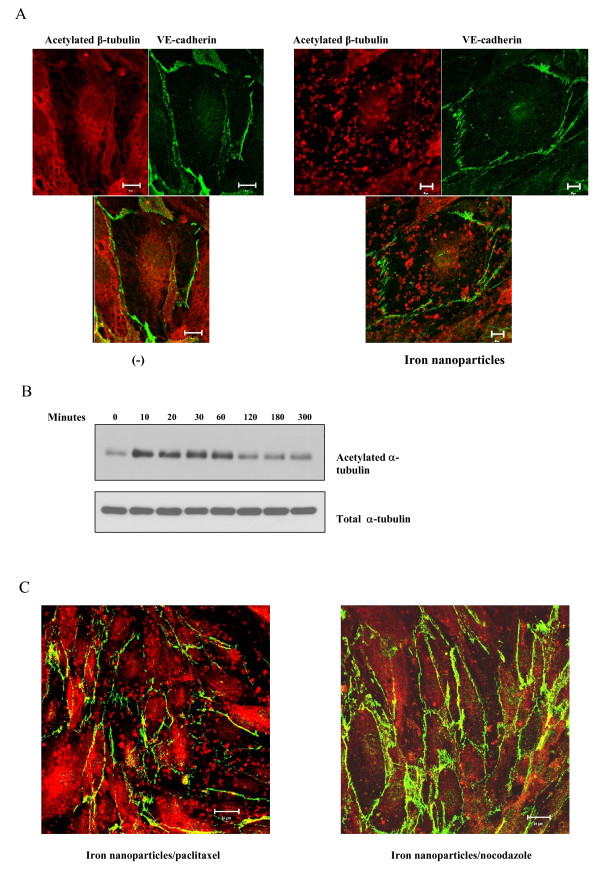
**Iron nanoparticles induce cell permeability through microtubule remodeling in HMVECs**. A, Iron nanoparticles induce microtubule remodeling and redistribution. HMVECs were grown on coverslips and serum-starved overnight. Iron nanoparticles (50 μg/ml) were used to stimulate the cells for 30 min. After exposure, the cells were fixed and stained for acetylated β-tubulin (red color) and VE-cadherin (green color). A Zeiss confocal microscope was applied to take the images. The bottom panels of each image are overlays of two different stains. The size of the scale bar is 20 μm. B, A time course of iron nanoparticle-induced microtubule stabilization. HMVECs were grown to a confluent monolayer and serum-starved overnight. The cells were exposed to 50 μg/ml iron nanoparticles for different periods of time as indicated. After exposure, the cells were lysed. The lysates were resolved on SDS-PAGE gel, and anti-acetylated α-tubulin antibody was used to detect acetylated α-tubulin. C, The monolayer HMVECs were pretreated with either 100 nM paclitaxel or 10 nM nocodazole for one hour as indicated, followed by exposure to 50 μg/ml iron nanoparticles for 30 min. The cells were fixed and stained for acetylated α-tubulin (red color) and VE-cadherin (green color). A Zeiss confocal microscope was applied to take the images. Each image is an overlay of two different stains. The size of the scale bar is 20 μm.

We next examined the underlying molecular mechanisms leading to an increase in permeability upon iron nanoparticle stimulation. Cytoskeleton protein microtubules are the major structural proteins involved in endothelial cell permeability through the dynamic remodeling processes [13,14]. This study sought to investigate the importance of microtubule remodeling in iron nanoparticle-induced cell permeability in HMVECs. Here, we first identified whether iron nanoparticles had an ability to induce microtubule remodeling in HMVECs. As shown in figure 3A, the cells treated with iron nanoparticles exhibited a significant remodeled microtubule structure. In normal HMVECs, the acetylated microtubules, a stabilized form of microtubules, formed network-fiber structures surrounding the nuclear area. However, upon exposure to iron nanoparticles, the network-fiber structures were disrupted to form dotted structures distributed evenly throughout the cells, and the amount of acetylated microtubules was also increased. Next, we investigated the effects of iron nanoparticles on acetylated microtubules with immunoblotting analysis. As shown in figure 3B, iron nanoparticles induced an increase in acetylated microtubules as early as 10 min after the treatment. The increase in acetylated microtubules was maintained up to 1 h. These results demonstrate that iron nanoparticle exposure promotes microtubule polymerization and altered distribution in HMVECs.

We then determined the involvement of microtubule remodeling in iron nanoparticle-induced cell permeability with different kinds of microtubule inhibitors: nocodazole and paclitaxel. Nocodazole depolymerizes microtubules and paclitaxel polymerizes microtubules. The results show that the pretreatment with paclitaxel increased iron nanoparticle-induced cell permeability whereas the pretreatment with nocodazole decreased the permeability (Figure 3C). Taken together, these results support the hypothesis that iron nanoparticles induce endothelial cell permeability in HMVECs through the stabilization of microtubule structures.

### Iron nanoparticles induce cell permeability through the production of ROS in HMVECs

**Figure 4:**
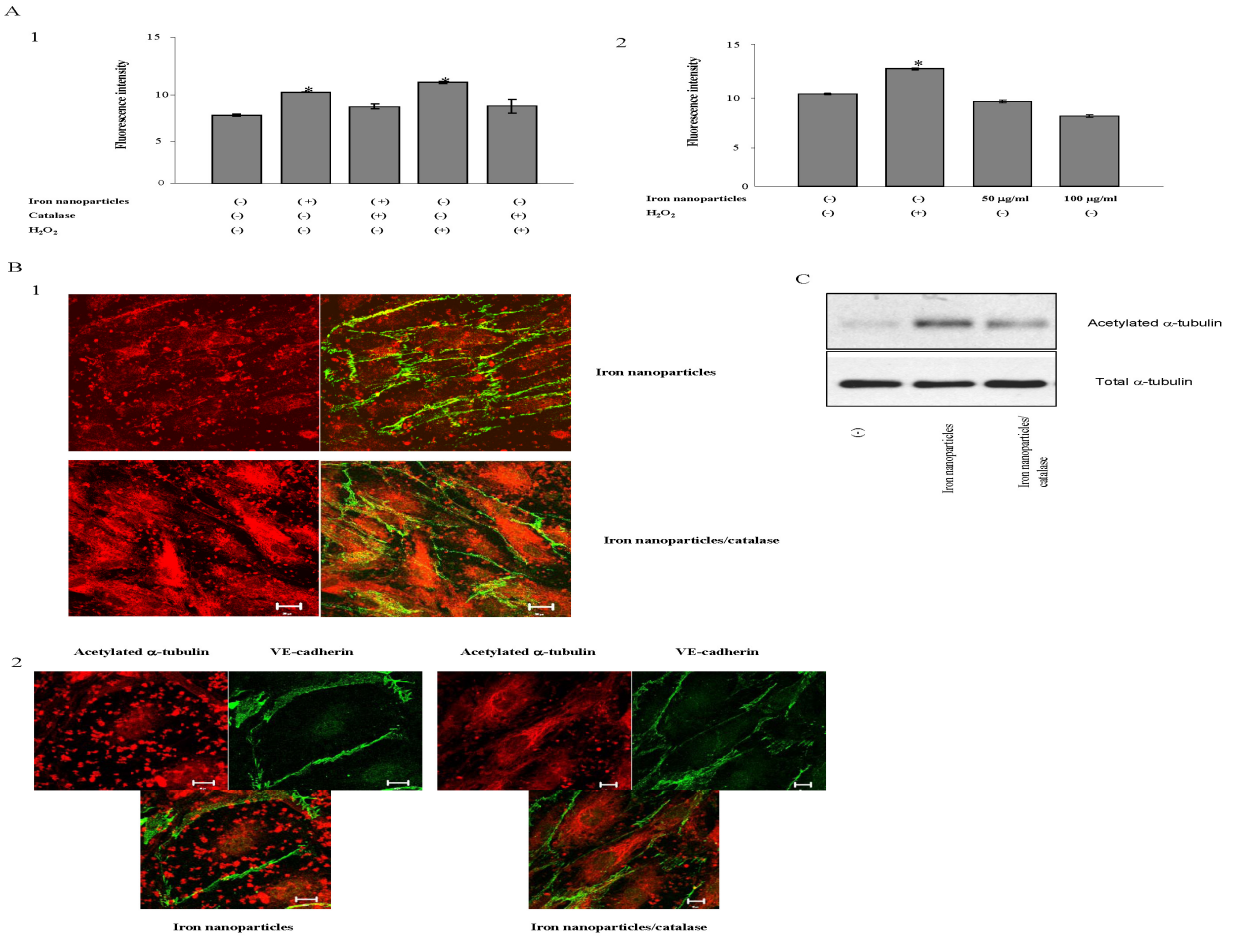
**ROS production is involved in iron nanoparticle-induced cell permeability**. A, Iron nanoparticles induce the production of ROS in HMVECs. 1. Iron nanoparticles induce the production of ROS in HMVECs. The monolayer HMVECs were pretreated with 10 μM CM-H_2_DCFDA, as well as 10,000 unit catalase as indicated, for one hour, followed by exposure to 50 μg/ml iron nanoparticles or 500 μM H_2_O_2 _for another hour as indicated. After the stimulation, the cells were collected and analyzed by a flow cytometry. Values given are means ± SD (t-test, n = 5, *p < 0.01). 2. Iron nanoparticles do not induce the production of ROS in cell-free system. The hydrolyzed CM-H_2_DCF-DA was mixed with 50 μg/ml iron nanoparticles, 100 μg/ml iron nanoparticles, or 1 mM H_2_O_2 _as indicated, followed by the fluorescence measurements using a cytoflour series 4000 plate reader. Values given are means ± SD (t-test, n = 5, *p < 0.05). B, Catalase pretreatment inhibits iron nanoparticle-induced microtubule remodeling and permeability in HMVECs. HMVECs were pretreated with 10,000 units/ml of catalase for one hour, followed by exposure to 50 μg/ml iron nanoparticles for 30 minutes. The cells were fixed and stained for acetylated α-tubulin (red color) and VE-cadherin (green color). A Zeiss confocal microscope was applied to take the images. The size of the scale bar is 20 μm. 1. Images of microtubule remodeling and cell permeability. The right panels are an overlay of two different stains. 2. Images of microtubule remodeling for the individual cells. C, Catalase pretreatment inhibits iron nanoparticle-induced microtubule stabilization. HMVECs were pretreated with 10,000 units/ml catalase for one hour, followed by the exposure to 50 μg/ml iron nanoparticles for one hour. The cells were lysed, and the lysates were resolved with 8% SDS-PAGE gel. An anti-acetylated α-tubulin antibody was applied to detect the expression of acetylated α-tubulin.

Accumulating evidence strongly suggest that many materials at the nanoparticle size have the ability to induce the production of ROS [15]. We sought to determine if the production of ROS is involved in iron nanoparticle-induced cell permeability. First, we examined whether iron nanoparticle exposure stimulated ROS production in HMVECs with flow cytometry analysis. As shown in figure 4A1, iron nanoparticle exposure significantly increased ROS production in 1 hour, compared to the unexposed cells. To determine the specificity of ROS production, the cells were pretreated with catalase, a ROS scavenger, followed by iron nanoparticle exposure. The results showed that catalase pretreatment blocked iron nanoparticle-induced ROS in HMVECs (Figure 4A1). We also exposed the cells to 500 μM H_2_O_2 _to set it as a positive control. Our results demonstrated that H_2_O_2 _exposure induced the production of ROS in HMVECs, which was significantly inhibited by catalase (Figure 4A1). To exclude the possibility that iron nanoparticles may generate ROS intrinsically, we measured the production of ROS in cell free systems. Our results found that iron nanoparticles were unable to produce ROS in cell-free systems (cell culture media without HMVECs); however the positive control, H_2_O_2_, was able to produce significant amount of ROS in the same systems (Figure 4A2). These results demonstrate that iron nanoparticle-induced ROS production in HMVECs is generated from the cell oxidative stress response. We then investigated the importance of ROS production in iron nanoparticle-induced cell permeability with a ROS scavenger catalase. As shown in figure 4B1 and 4B2, pretreatment of cells with catalase inhibited iron nanoparticle-induced dotted microtubule structures and microtubule distribution, and inhibited cell permeability as well, indicating that ROS are involved in the regulation of iron nanoparticle-induced microtubule remodeling and cell permeability. This study further confirmed the effect of ROS production on microtubule remodeling by immunoblotting analysis. As shown in figure 4C, the pretreatment of cells with catalase significantly attenuated iron nanoparticle-induced acetylated microtubule formation.

### Iron nanoparticles induce HMVEC permeability via GSK-3β signaling pathways

**Figure 5:**
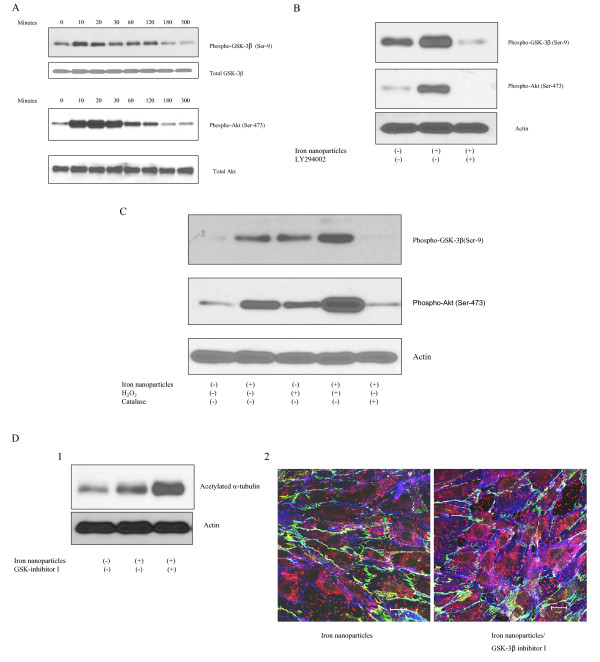
**GSK-3β signaling pathways are involved in iron nanoparticle-induced endothelial cell permeability**. A, Iron nanoparticle treatment induces the inhibition of GSK-3β and the activation of Akt. HMVECs were grown to a confluent monolayer and serum-starved overnight. The cells were exposed to 50 μg/ml iron nanoparticles for different periods of time as indicated. After the stimulation, the cells were lysed. The lysates were resolved on SDS-PAGE gel, followed by probing with different antibodies as indicated. B, PI3K is involved in iron nanoparticle-induced Akt activation and GSK-3β inhibition. HMVECS were pretreated with 10 μM LY294002, a PI3K inhibitor, for one hour, and then exposed to 50 μg/ml iron nanoparticles for 30 minutes, followed by Western blot analysis. C, ROS production is involved in iron nanoparticle-induced Akt activation and GSK-3β inhibition. HMVECs were pretreated with either 200 μM hydrogen peroxide (H_2_O_2_) or 10,000 units/ml catalase for one hour, and then exposed to 50 μg/ml iron nanoparticles for 30 min, followed by Western blot analysis. D, The inhibition of GSK-3β enhances iron nanoparticle-induced microtubule remodeling and cell permeability in HMVECs. HMVECs were pretreated with 5 μM GSK-3β inhibitor I for one hour, and then exposed to 50 μg/ml iron nanoparticles for 30 minutes, followed by Western blot analysis (1) and confocal microscopy analysis (2). Blue color stains for actin filaments, red color stains for acetylated α-tubulin, and green color stains for VE-cadherin. The size of the scale bar is 20 μm.

The inhibition of GSK-3β (phosphorylation at serine 9) plays a major role in regulating microtubule stabilization [16]. This study investigated if GSK-3β is involved in iron nanoparticle-induced microtubule stabilization and cell permeability in HMVECs. As shown in figure 5A, iron nanoparticles induced serine-9 phosphorylation of GSK-3β within 10 min after the treatment, and the increase in the phosphorylation was maintained up to 2 h. We then explored the activities of Akt, an upstream kinase of GSK-3β, upon iron nanoparticle exposure. It was found that the pattern of Akt phosphorylation (activation) was the same as that of GSK-3β (Ser-9) phosphorylation (Figure 5A). The results also showed that iron nanoparticle-induced phosphorylation of both GSK-3β (Ser-9) and Akt was dramatically attenuated by the pretreatment with a PI3K inhibitor, LY294002 (Figure 5B). These results indicate that iron nanoparticles have an ability to induce the inhibition of GSK-3β through the PI3K/Akt signaling pathway.

We then determined the role of ROS production in iron nanoparticle-induced GSK-3β inhibition and Akt activation. As shown in figure 5C, hydrogen peroxide alone increased the phosphorylation of both GSK-3β (Ser-9) and Akt (Ser -473) in a manner similar to that of iron nanoparticle exposure. When the cells were treated with iron nanoparticles plus hydrogen peroxide, the induction of GSK-3β (Ser-9) and Akt (Ser-473) phosphorylation was enhanced compared with either treatment alone. Furthermore, the pretreatment of cells with catalase attenuated iron nanoparticle-induced phosphorylation of both GSK-3β (Ser 9) and Akt (Ser 473) (Figure 5C). These results indicate that ROS production plays a regulatory role in iron nanoparticle-induced GSK-3β inhibition and Akt activation.

Lastly, this study sought to determine the regulatory roles of GSK-3β inhibition in iron nanoparticle-induced microtubule remodeling and cell permeability by using a pharmacological inhibitor of GSK-3β, GSK-3β inhibitor I. These results demonstrate that the pretreatment with GSK-3β inhibitor I enhanced iron nanoparticle-induced microtubule remodeling and cell permeability (Figure 5D1 and 5D2). These results suggest that GSK-3β may be involved in regulating iron nanoparticle-induced microtubule remodeling and cell permeability in HMVECs.

## Discussion

The endothelial cells line the luminal surface of blood vessels to form a semi-permeable barrier to regulate vascular tone, blood fluidity, angiogenesis, and extravasation of blood components and other substances [3,17]. The changes in this semi-permeable barrier are critical in controlling the passage of macromolecules and fluid from the blood circulation into tissues, which is a key molecular process for drug delivery, as well as for the pathogenesis of inflammatory diseases, cardiovascular diseases, lung injury, carcinogenesis [3,17]. The endothelial semi-permeable barrier controls the transfer of many soluble and insoluble substances via two pathways: transcellular and paracellular pathways [3]. The transcellular pathway transports many substances via transcytosis in vesicle carriers whereas the paracellular pathway transfers substances through tightly linked inter-endothelial junctions. The paracellular pathway-mediated permeability is maintained by an equilibrium between cytoskeleton-generated contractile force and cell-cell junction and contact-induced adhesive force [18]. Any shift in this equilibrium will results in the opening and closing of paracellular pathways to affect the transport of macromolecules and drugs [18]. The unperturbed endothelium paracellular pathway can only allow transport of molecules with a radius of less than 3 nm to move passively across the barrier [3]. However, in response to stimulation or pathologic conditions, the paracellular pathway becomes leaky, opening inter-endothelial junctions to form the gaps between endothelial cells to allow the translocation of larger molecules [19]. Previous studies reported that nanoparticles are able to across the semi-permeable barrier via transcellular pathways [20]. However, little is known about the effects of nanoparticles on endothelial paracellular pathways. In this study, we demonstrate that iron nanoparticle stimulation induced an increase in cell permeability *in vitro*, i.e., the formation of gap structures between endothelial cells in a confluent endothelial monolayer. This indicates that the exposure to iron nanoparticles may be able to facilitate extravasation of macromolecules and drugs, as well as nanoparticle themselves, into surrounding tissues. The results obtained from this study provide a new insight on the effects of nanoparticles on vascular transport of drugs and macromolecules.

The concentration (50 μg/ml) of iron nanoparticles applied in this manuscript is relevant to the dosages employed in current clinical trials. Human imaging in central nervous system and in carotid atherosclerotic plague utilized iron nanoparticles at 45 μmol Fe/kg for magnetic resonance imaging [21], which is about the concentration of 70 μg/ml (at average 50 kg human weight and 5000 ml human blood volume). Rat clinical trials for magnetic resonance imaging injected iron nanoparticles via intravenous injection at the dose of 5 mg Fe/kg [22], which is about the concentration of 66 μg/ml (at average 200 g rat weight and 15 ml rat blood volume). Therefore, our concentration (50 μg/ml) of iron nanoparticles is achievable in the circulation. The studies of iron nanoparticle pharmacokinetics and biodistribution demonstrated that the high doses of iron nanoparticles are needed to reach deep compartments of bodies in clinical imaging experiments [21].

The cellular uptake profiling shows that the peak of uptake occurs within 30 min of the stimulation and only 10% of the cells still retain iron nanoparticles after one hour of exposure. However, the alteration of signaling transduction pathway are maintained for almost two hours following exposure to iron nanoparticles, and both our confocal microscope analysis and ECIS assays show that iron nanoparticle-induced permeability lasts at least 5 hours after the stimulation. There results indicate that exposure of HMVECs to iron nanoparticles induces a prolong alteration of endothelial monolayer barrier function.

The unique features of nanoparticles are small particle size and large surface area, which exposes atoms or molecules on the particle surface instead of covering them within the interior of the material [15]. Accumulating evidence shows that nanoparticle-induced ROS oxidant stress response is the major mechanism for the induction of various biological effects [15,23]. At the low basal level, ROS is involved in regulating normal cell functions; however, at a higher abnormal level, ROS induce cell injury and death [24]. In this study, it was found that the exposure to iron nanoparticles induces the production of ROS in HMVECs. Furthermore, we found that the addition of H_2_O_2 _enhances iron nanoparticle-induced cell permeability whereas the elimination of ROS with catalase abrogates iron nanoparticle-induced cell permeability, demonstrating that the production of ROS is involved in iron nanoparticle-induced permeability. Our results regarding the roles of ROS in endothelial cell permeability are consistent with several published observations [24]. Numerous studies have shown that ROS-induced oxidant stress directly increases endothelial permeability [6,24]. The treatment of endothelial cell monolayers with ROS generators, xanthine/xanthine oxidase or glucose/glucose oxidase, increases endothelial cell permeability in a dose dependent manner [25,26].

In this study, it was found that iron nanoparticle-induced ROS production may regulate cell permeability through the remodeling of microtubules in HMVECs. It was well established that microtubules remodeling is closely related to the changes in endothelial cell permeability [3,19]. However, it is controversial as to the mechanisms by which microtubule remodeling regulates cell permeability. Several previous studies showed that microtubules are involved in regulating both tumor necrosis factor-α and thrombin-induced endothelial permeability through destabilization (depolymerization) while some observations found that microtubules modulate cell permeability via stabilization (polymerization) in tumor cells [14,27,28]. In this study, it is demonstrated that iron nanoparticle exposure induces both polymerization and redistribution of microtubules through the production of ROS in HMVECs. Furthermore, it is demonstrated that ROS-mediated microtubule remodeling is involved in iron nanoparticle-induced endothelial cell permeability. The finding that iron nanoparticle exposure stimulates the activation of PI-3 kinase/Akt/GSK-3β signaling pathways further supports our observations of iron nanoparticle-induced microtubule remodeling. GSK-3β is a key kinase that regulates microtubule depolymerization via the phosphorylation of several microtubule-associated proteins [29]. Serine-9 phosphorylation of GSK-3β by Akt inhibits its activities, which abrogates GSK-3β's ability to phosphorylate microtubule-associated protein and results in microtubule stabilization [29]. These results demonstrated that iron nanoparticle exposure induces the activation of Akt and inhibition of GSK-3β in a PI3-kinase dependent manner, and both our inhibitory and stimulatory assays strongly indicate that Akt/GSK-3β signaling pathways are involved in iron nanoparticle-induced cell permeability through ROS-mediated remodeling of microtubules.

**Figure 6:**
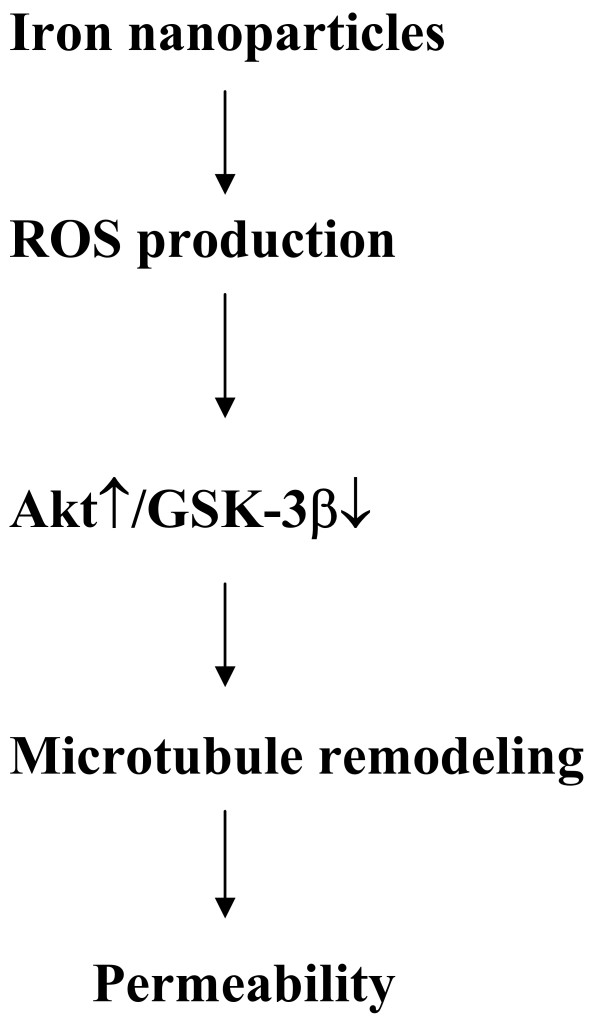
**Schematic representation of signal transduction from iron nanoparticle stimulation to cell permeability**.

Taken together, these results demonstrate that exposure to iron nanoparticle induces an increase in endothelial cell permeability through ROS oxidative stress- modulated microtubule remodeling (Figure 6). The findings from this study provide new insights on the effects of nanoparticles on vascular transport of macromolecules and drugs. Results provided here may have implications for understanding the bioactivity of engineered nanoparticles, which can inform potential applications in both nanomedicine and elucidate nanotoxicology in general.

## Methods

### Reagents

Cell culture medium EBM-2 was obtained from Lonza (Boston, MA). Fetal bovine serum was obtained from Atlanta Biologicals (Lawrenceville, GA). Fe_2_O_3 _nanoparticles were purchased from nGIMAT (Atlanta, GA). Acetylated-tubulin antibody, total tubulin antibody, actin antibody, catalase, hydrogen peroxide, EGF growth supplement, and hydrocortisone were from Sigma (St. Louis, MO). Protease and phosphatase inhibitor cocktail was from Pierce (Rockford, IL). LY294002, GSK-3β inhibitor I, nocodazole and paclitaxel were obtained from Calbiochem (La Jolla, CA). Penicillin and Streptomycin antibiotics, 5-(and-6)-chloromethyl-2',7'-dichlorodihydrofluorescein diacetate, acetyl ester (CM-H_2_DCFDA), secondary antibodies-conjugated with FITC, TRITC, and Cy5 were purchased from Invitrogen (Eugene, OR). Phospho-Akt (ser-473) and total Akt antibodies were from Cell Signaling Technology (Boston, MA). Phospho-GSK-3β (ser-9) and total GSK-3β antibodies were from Santa Cruz Biotechnology (Santa Cruz, CA). VE-Cadherin antibody was purchased from Alexis (San Diego, CA).

### Cell culture

The human microvascular endothelial cells (HMVECs) were a kind gift from Dr. Rong Shao (Biomedical Research Institute, Baystate Medical Center/University of Massachusetts at Amherst, Springfield, MA, USA). The cells were cultured according to the protocol described previously [11]. Briefly, HMVEC were grown in endothelial basal medium-2 (EBM-2) (Lonza, Boston MA) supplemented with 10% (v/v) fetal bovine serum (FBS) (Atlanta Biologicals, Lawrenceville, GA), 100 U/ml penicillin and 10 μg/ml streptomycin, 1 μg/ml of epidermal growth factor and 50 μg/ml hydrocortisone. The cells were maintained in an incubator at 37°C with 5% CO_2_.

### Particles preparation and size measurements

Fe_2_O_3 _nanoparticles were purchased from nGIMAT (Atlanta, GA). Their surface area was approximately 165 m2/g, the average powered particle size was <10 nm, and there were trace amounts of Lead and Bismuth potentially in the particles. The iron nanoparticles were suspended in 0.1% fetal bovine serum (FBS) cell culture media at a concentration of 2.5 mg/ml. Once the nanoparticles were dispersed in 0.1% FBS cell culture media, the suspension was indirectly sonicated at 4°C for 10 min with a Hielscher-Ultrasound Technology Sonicator (UIS 259L) at amplitude 100% and cycle 1. After the indirect sonication, the suspension was further directly sonicated at 4°C for 5 min at a duty cycle setting of 10% and output of 5 with a Branson 450 Sonifier probe sonicator. The stock solution (2.5 mg/ml) of iron nanoparticles was kept at 4°C and used within 2 weeks for the experiments. The working concentration of iron nanoparticle was 50 μg/ml. Prior to being diluted to the working concentration, the stock solution was directly sonicated at 4°C for 1 min at the setting indicated above. The particle size was determined by both dynamic light scattering using Nanotrac 252 (Microtrac, Montgomeryville, PA) [30] and by a transmission electron microscope.

### Lactate dehydrogenase (LDH) assay

The LDH release assays were measured using the LDH assay kit form Roche Diagnostics Inc. (Indianapolis, IN) according to the manufacturer instructions.

### ROS production in Cell-free system

The measurements of ROS production in cell-free system were performed according to the previously published methods[31]. Briefly, **7**.5 μl of 10 mM CM-H_2_DCF-DA was chemically hydrolyzed to CM-H_2_DCF in 1 ml of 0.01 N NaOH in dark for 30 min at the room temperature, followed by neutralizing with 0.5 ml of 0.1 M PBS (pH 7.4). The reaction mixture was freshly prepared by mixing 400 μl of the neutralized CM-H_2_DCF, 1.6 ml of EBM-2 medium (0.1% FBS), and 0.2 units of horse radish peroxidase (HRP) to obtain a final concentration of 10 μM CM-H_2_DCF. Then, 2 μl of iron nanoparticle stock solution (25 mg/ml) or 2 μl of H_2_O_2 _stock solution (0.5 M) was added into 2 ml of the reaction mixture to make a final concentration of 50 μg/ml iron nanoparticles or 500 μM H_2_O_2_, respectively. After 30 minute incubation, fluorescence generated from the oxidation of CM-H_2_DCF to DCF was measured using a cytoflour series 4000 plate reader (PerSeptive Biosystems, Inc., Framingham, MA) at 485 nm excitation and 530 emission

### ROS measurements by flow cytometry

ROS measurements by flow cytometry analysis were performed according to the methods described previously [32]. HMVECs were pretreated with 10 μM CM-H_2_DCFDA for 60 min. After the pretreatment, the cell culture media was removed and replaced with the media containing iron nanoparticles (50 μg/ml) and 10 μM CM-H_2_DCFDA for further stimulation. After the stimulation, the cells were quenched on ice for 10 min then washed three times with ice-cold PBS before they were harvested by scrapping. The cells were fixed with 10% formaldehyde for 20 min at room temperature and then washed three times with PBS, followed by resuspension in 400 ml of PBS. ROS measurements were carried out by a flow cytometry using FACSCalibur system (BD Biosciences, Rutherford, NJ) with a 488-nm excitation beam. The signals were obtained using a 530-nm band-pass filter for CM-H_2_DCFDA. Each measurement was based on the mean fluorescence intensity of 10,000 cells.

### Transendothelial electrical resistance

The transendothelial electrical resistance (TER) was measured using electrical cell-substrate impedance sensing system (ECIS) (Applied Biophysics, Troy, NY) according to the published protocol [33]. Briefly, HMVECs were grown to confluent monolayer on ECIS culture ware and serum-starved overnight. The electrical resistance was measured on cells located on the small gold electrodes in each of the wells. The culture medium was the electrolyte. The small gold electrode covered by confluent HMVECs and a larger gold counter electrode were connected to a phase-sensitive lock-in amplifier. A constant current of 1 μA was supplied by a 1-V, 4,000-Hz alternating current through a 1-MΩ resistor. Changes in voltage between the small electrode and the large counter electrode were continuously monitored by the lock-in amplifier, stored, and then calculated as resistance.

### Immunofluorescence assay and Western blot analysis

Immunofluorescence assays were performed according to the methods published previously [34]. Briefly, HMVECs were grown on coverslides. After treatment, cells were fixed and permeabilized, followed by labeling with the specific antibodies for the targeted proteins as well as immunofluorescence-conjugated secondary antibodies. The labeled coverslides were mounted to the slides with antifade reagent (Invitrogen, Eugene, OR). A Zeiss LSM 510 microscope was used to obtain images. Scale bars were generated and inserted by LSM software. Western blot analysis was performed according to the methods described previously [35]. Briefly, the cell lysates were resolved in 8% SDS-PAGE gel, and then transferred to PVDF membranes, followed by blotting with different antibodies for the individual targeted proteins. Horseradish peroxidase-conjugated secondary antibodies (GE Healthcare) were applied to visualize proteins using chemiluminescence.

### Transmission electron microscopy (TEM) of iron nanoparticles

TEM of iron nanoparticles was performed according to previously published procedures [36]. Briefly, HMVECs were grown and stimulated in transwell tissue polycarbonate membrane polystyrene plates, and were then washed with ice cold PBS. The cells were fixed in Karnovsky's fixative (2.5% glutaraldehyde + 3% paraformaldehyde in 0.1 M sodium cacodylate, pH 7.4), and then washed three times in 0.1 M sodium cacodylate and post-fixed in 1% osmium tetra oxide, followed by washing with 0.1 M sodium cacodylate and distilled water. The cells were dehydrated by sequential washings in 25%, 50% and 100% ethanol then embedded in LX-112 (Ladd, Williston, VT). The ultrathin sections were stained with uranyl acetate and lead citrate and examined with a TEM (JEOL 1220, Tokyo, Japan). To measure the size distribution of iron nanoparticles in the cell culture medium, iron nanoparticles (50 μg/ml) were prepared as indicated above. An aliquot of this working solution was then dropped on a formvar-coated grid, let to dry then analyzed by transmission electron microscopy.

### Dynamic light scattering measurements

Suspension of iron nanoparticles at 50 μg/ml was prepared in 0.1% FBS EBM-2 media. The iron nanoparticle suspension was sonicated with a probe sonicator (Branson Sonifier 450, 10 W continuous output) for 30 min and then vortexed for 1 min, followed by measuring the particle size by dynamic light scattering using Nanotrac 252 (Microtrac, Montgomeryville, PA). During sonication, heat was dissipated by placing the samples on ice [30].

## Abbreviations

HMVECS: human microvascular endothelial cells; ROS: reactive oxygen species; TEM: transmission electron microscopy; DLA: dynamic light scattering.

## Competing interests

The authors declare that they have no competing interests.

## Authors' contributions

PLA was substantially involved in conducting the experiments and drafting the manuscript. YQ is one of the project leaders and was substantially involved in the design of the project and drafting the manuscript. NLG, RS, XS, VV, and VC participated in the design of the study and revised the manuscript. MP was involved in the particle preparation. DSB carried out the electron microscopy analysis. DP performed dynamic light scattering measurements. DCF is one of the project leaders and is involved in the design of the project.

## Disclaimer

The findings and conclusions in this report are those of the author(s) and do not necessarily represent the views of the National Institute for Occupational Safety and Health.
